# Synaptic alterations and neuronal firing in human epileptic neocortical excitatory networks

**DOI:** 10.3389/fnsyn.2023.1233569

**Published:** 2023-08-10

**Authors:** Réka Bod, Kinga Tóth, Nour Essam, Estilla Zsófia Tóth, Loránd Erõss, László Entz, Attila G. Bagó, Dániel Fabó, István Ulbert, Lucia Wittner

**Affiliations:** ^1^Institute of Cognitive Neuroscience and Psychology, Research Centre for Natural Sciences, Eötvös Loránd Research Network, Budapest, Hungary; ^2^Semmelweis University Doctoral School, Budapest, Hungary; ^3^National Institute of Mental Health, Neurology and Neurosurgery, Budapest, Hungary; ^4^Faculty of Information Technology and Bionics, Pázmány Péter Catholic University, Budapest, Hungary

**Keywords:** excitatory system, epilepsy, human neocortex, single unit activity, synchronous population activity, synaptic reorganization

## Abstract

Epilepsy is a prevalent neurological condition, with underlying neuronal mechanisms involving hyperexcitability and hypersynchrony. Imbalance between excitatory and inhibitory circuits, as well as histological reorganization are relatively well-documented in animal models or even in the human hippocampus, but less is known about human neocortical epileptic activity. Our knowledge about changes in the excitatory signaling is especially scarce, compared to that about the inhibitory cell population. This study investigated the firing properties of single neurons in the human neocortex *in vitro*, during pharmacological blockade of glutamate receptors, and additionally evaluated anatomical changes in the excitatory circuit in tissue samples from epileptic and non-epileptic patients. Both epileptic and non-epileptic tissues exhibited spontaneous population activity (SPA), NMDA receptor antagonization reduced SPA recurrence only in epileptic tissue, whereas further blockade of AMPA/kainate receptors reversibly abolished SPA emergence regardless of epilepsy. Firing rates did not significantly change in excitatory principal cells and inhibitory interneurons during pharmacological experiments. Granular layer (L4) neurons showed an increased firing rate in epileptic compared to non-epileptic tissue. The burstiness of neurons remained unchanged, except for that of inhibitory cells in epileptic recordings, which decreased during blockade of glutamate receptors. Crosscorrelograms computed from single neuron discharge revealed both mono- and polysynaptic connections, particularly involving intrinsically bursting principal cells. Histological investigations found similar densities of SMI-32-immunopositive long-range projecting pyramidal cells in both groups, and shorter excitatory synaptic active zones with a higher proportion of perforated synapses in the epileptic group. These findings provide insights into epileptic modifications from the perspective of the excitatory system and highlight discrete alterations in firing patterns and synaptic structure. Our data suggest that NMDA-dependent glutamatergic signaling, as well as the excitatory synaptic machinery are perturbed in epilepsy, which might contribute to epileptic activity in the human neocortex.

## 1. Introduction

Epilepsy is one of the most common neurological disorders in humans, and it is thought to be related to hyperactivity of neuronal networks (Fisher et al., 2014). The imbalance between excitatory and inhibitory circuits is the most well-known hypothesis explaining the generation of epileptic paroxysmal activities, i.e., seizures and interictal spikes. The excess excitation – linked to the generation of epileptic activity – is usually provided by the enhancement of excitatory signaling together with a loss of inhibition. Increased excitatory processes were connected to the generation of seizures with a hypersynchronous onset (Avoli et al., 2016), which seizure types are characteristic to patients with temporal lobe epilepsy (Perucca et al., 2014). Strong and efficient excitatory synaptic transmission resulting from the bursting behavior and the paroxysmal depolarization shift of pyramidal cells was connected to the generation of interictal spikes in several *in vitro* (pharmacological) models in the human neocortex (for review see de Curtis and Avanzini, 2001). Changes in the inhibitory system in the epileptic human neocortex are not uniform: a decreased inhibitory but not excitatory firing was demonstrated in the hippocampus of epileptic patients prior neocortical seizures invading the medial temporal lobe (Misra et al., 2018), whereas an increased inhibitory neuron firing was found at the beginning of low voltage fast onset seizures (Dehghani et al., 2016; Elahian et al., 2018), the most frequently occurring seizure type in neocortical epilepsies (Avoli et al., 2016). The selective activation of the perisomatic inhibitory neurons induced seizures in animals (Levesque et al., 2022), while a compromised inhibition was shown to account for the spread of seizures through large neocortical areas in humans (Trevelyan et al., 2015). Thus, several lines of evidence indicate that a more efficient excitation together with a perturbed inhibition can lead to the development of epileptic activity in neocortical networks.

Signs of hyperexcitability and epileptic reorganization have been shown in the human epileptic neocortex both at cellular and network levels. Synchronous population bursts – thought to be the correlates of interictal spikes – arise *in vitro*, in a physiological perfusion bath in neocortical (Köhling et al., 1998; Gorji et al., 2002; Roopun et al., 2010; Tóth et al., 2018; Kandrács et al., 2019) and hippocampal (Cohen et al., 2002; Wozny et al., 2005; Huberfeld et al., 2007, 2011; Wittner et al., 2009) tissue derived from epileptic patients. Similar population events were detected in neocortical slice preparations from non-epileptic tumor patients as well (Kerekes et al., 2014; Tóth et al., 2018; Kandrács et al., 2019), and correlated intraoperative electrocorticography to *in vitro* electrophysiology using the same neocortical tissues demonstrated that these synchronies appear in samples derived from non-epileptic patients, from brain areas without any signs of epileptic activity as well, thus, cannot be related to epilepsy (Hofer et al., 2022). The synchronous population activity (SPA) emerging in neocortical slices exhibited higher excitability in epileptic compared to non-epileptic tissue (Tóth et al., 2018; Hofer et al., 2022). This manifested in higher occurrence rate, larger amplitude, and more depolarizing pyramidal cells, firing more reliably during population events, pointing to the crucial role of pyramidal cells in the generation of synchronous activities. In intracellular records, SPA was reflected as large, and sometimes complex postsynaptic potentials (Schwartzkroin and Knowles, 1984; McCormick, 1989; Pallud et al., 2014), similar to the complex synaptic events evoked by activating single pyramidal cells in the human neocortex of non-epileptic patients (Molnár et al., 2008; Szegedi et al., 2016). The generation of these population events involves both the GABAergic and glutamatergic systems (Schwartzkroin and Haglund, 1986; Köhling et al., 1998; Cohen et al., 2002; Tóth et al., 2018), and evidences are provided for the leading role of the excitatory (Williamson et al., 2003; Kandrács et al., 2019) and the inhibitory (Cohen et al., 2002; Pallud et al., 2014) cells in initiating these synchronies in the human neocortex and hippocampus, respectively.

Neocortical excitatory pyramidal cells with subcortical axonal projections are mainly located in layers III, V, and VI, and can be stained by the SMI-32 antibody recognizing the non-phosphorylated neurofilament (Sternberger and Sternberger, 1983). These pyramidal neurons were found to be highly vulnerable in several neurological conditions including Alzheimer’s and Huntington’s diseases, acute and chronic ischemia (Cudkowicz and Kowall, 1990; Hof et al., 1990; Leifer and Kowall, 1993). In focal cortical dysplasia, the number of SMI-32-positive cells has decreased, and their morphology has also been changed, their dendrites became shorter (Fauser et al., 2013). Human neocortical pyramidal neurons show extensive histological changes in epilepsy, affecting their morphology (Alonso-Nanclares et al., 2005), their receptor- (Wierschke et al., 2010), and transporter content (Aronica et al., 2007) as well. Inhibitory cells are also modified in neocortical epilepsies. The heterogeneity of the inhibitory interneurons (INs) is linked to heterogeneous anatomical changes, but the same interneuron type can also show different anatomical modifications depending on the epilepsy type examined. For example, parvalbumin-positive perisomatic inhibitory cells were found to decrease in number in the epileptic peritumoral neocortex (Marco and DeFelipe, 1997) and cortical dysplasia (Spreafico et al., 1998), but were preserved in microdysgenesis and cortical tuber associated epilepsy (Valencia et al., 2006). Although interneurons show very complex and different histological changes, all research groups agree that the inhibitory circuit is perturbed, and this might contribute to the generation of epileptic activity.

Despite the general neocortical cell loss observed in epilepsy (Thom et al., 2005), the density of excitatory synapses was found to be increased, either with decreased (Marco and DeFelipe, 1997) or with unchanged (Tóth et al., 2018) density of inhibitory synapses in epileptic patients. In cortical areas axospinous synapses are specialized for excitatory neurotransmission, which is realized through ionotropic glutamate receptors (GluRs). AMPA, i.e., α-amino-3-hydroxy-5-methyl-4-isoxazolepropionic acid-type GluRs are known to generate fast excitatory postsynaptic potentials, whereas N-methyl-D-aspartate (NMDA)-type receptors are thought to be involved in synaptic plasticity, the neuronal form of memory formation (for review see Traynelis et al., 2010). The growth of excitatory axons is coupled to spine division and to the enhancement of synaptic transmission (Harris et al., 1992). Perforated synapses are considered to be the intermediate form during the process of synapse division (Carlin and Siekevitz, 1983) and synapse turnover (Nieto-Sampedro et al., 1982). The presence of perforated synapses was also associated with synaptic plasticity (Geinisman et al., 1993) resulting from several forms of increased synaptic activity, such as visual training (Vrensen and Cardozo, 1981) or long-term potentiation (Geinisman et al., 1991; Harris et al., 2003). Pathological conditions, such as lesion- (Jones, 1999) or epilepsy induced axonal sprouting (Frotscher et al., 2006) lead to the increase in the number of perforated synapses. The increased synaptic transmission provided by the sprouting of excitatory axons most probably accounts for the hyperexcitability seen in epileptic cortices.

In this study, we investigated the activity of human neocortical single neurons in relation to excitatory glutamatergic signaling in an *in vitro* model spontaneously generating non-epileptic SPA. The firing properties of regular spiking and intrinsically bursting pyramidal cells and inhibitory neurons were assessed, in tissue samples derived from both epileptic and non-epileptic patients. We completed these analyses with anatomical examinations including the density SMI-32-stained neurons, as well as the synaptic length and postsynaptic target distribution of excitatory synapses. We conclude that the generation of SPA events in human neocortical slices depends on glutamate signaling through AMPA/kainate receptors, both in non-epileptic and epileptic tissues, whereas blockade of NMDA receptors decreases the recurrence frequency of SPAs only in epileptic tissue. The presence of different cell types during pharmacological application and their firing properties or burstiness suggest complex synaptic interactions in the human neocortex, which is simultaneously supported by anatomical investigations. We demonstrate the intense reorganization of the excitatory circuit in the human epileptic neocortex, manifesting in axonal sprouting, synapse division, as well as the specific target selection of the newly formed synapses. In summary, epileptic reorganization largely affects the glutamatergic circuitry in the human neocortex.

## 2. Materials and methods

### 2.1. Patients

The study involved patients who were undergoing brain surgery at the National Institute of Mental Health, Neurology and Neurosurgery in Budapest, Hungary. These patients provided their informed consent in writing, and the study was authorized by the Regional and Institutional Committee of Science and Research Ethics of the Scientific Council of Health (ETT TUKEB 20680-4/2012/EKU, IV/8358- 3/2021/EKU) and conducted in accordance with the Declaration of Helsinki. It should be noted that the patients included in this study were also involved in previous studies (Tóth et al., 2018; Kandrács et al., 2019; Hofer et al., 2022).

#### 2.1.1. Epileptic patients

Seven epileptic patients were included in this study, all suffering from pharmacoresistant epilepsy (Table 1). One patient had a tumor (ganglioglioma), two patients suffered from focal cortical dysplasia, three from hippocampal sclerosis, and one had a double pathology comprising both focal cortical dysplasia and hippocampal sclerosis. One epileptic sample derived from the frontal lobe, all others from the temporal lobe. The patients suffered from focal cortical epilepsy for 24 ± 20 years on average (mean ± SD).

**TABLE 1:** Patient data.

Patient code	Gender	Age	Diagnosis	Epilepsy time span (years)	Stage	Resected region	Seizure onset zone	Distance from tumor	Anatomy of obtained tissue
E2	F	21	Focal cortical dysplasia with glioneural heterotopia	2	Epi	Frontal	+	–	Dysgenetic
E4	M	24	Ganglioglioma grade I	4	Epi	Temporal	−	Distant	Normal
E5	F	18	Focal cortical dysplasia II B	5	Epi	Temporal	+	–	Dysgenetic
E6	M	51	Hippocampal sclerosis	50	Epi	Temporal	+	–	Normal
E12	M	40	Hippocampal sclerosis	35	Epi	Temporal	+	–	Normal
E13	F	53	Hippocampal sclerosis	40	Epi	Temporal	−	–	Normal
E15	M	35	Focal cortical dysplasia and hippocampal sclerosis	34	Epi	Temporal	+	–	Normal
T6	M	31	Cavernoma, hematoma intracerebralis acuta	–	No	Frontal	N/A	Distant	Normal
T7	F	58	Glioblastoma multiforme, meningitis	–	No	Temporal	N/A	Close	Infiltrated
T8	F	78	Glioblastoma multiforme, astrocytoma grade IV	–	No	Temporal	N/A	Distant	Normal
T17	F	74	Glioblastoma, with oligodendroglioma fragments, grade IV	–	No	Frontal	N/A	Close	Normal
T20	F	59	Breast carcinoma metastasis	–	No	Frontal	N/A	Close	Infiltrated
T21	F	69	Kidney carcinoma metastasis	–	No	Parietal	N/A	Distant	Normal

F, female; M, male; Epi, pharmacoresistant epilepsy; No, no epilepsy; Distant, >3 cm; Close, <3 cm.

#### 2.1.2. Non-epileptic patients

Six patients diagnosed with brain tumor were included in this study. Based on their anamnesis none of them had clinical or electrographic signs of epileptic seizures or interictal spikes before the date of their brain surgery. One patient had cavernoma, three had ganglioglioma, and two suffered from carcinoma metastasis (see Table 1 for details). We received samples from the temporal (*n* = 2), frontal (*n* = 3), and the parietal (*n* = 1) lobes. The distance of the obtained tissue from the tumor was provided by the neurosurgeon, based on magnetic resonance images.

### 2.2. Tissue sample handling

Tissue was transported from the operating room to the laboratory (located in the same building) in a cold, oxygenated solution containing (in mM) 248 D-sucrose, 26 NaHCO_3_, 1 KCl, 1 CaCl_2_, 10 MgCl_2_, 10 D-glucose, and 1 phenol red, equilibrated with 5% CO_2_ in 95% O_2_. Neocortical slices of 500 μm thickness were cut with a Leica VT1000S vibratome (Leica GmBH, Wetzlar, Germany, RRID:SCR_016495). They were transferred and maintained at 35–37°C in an interface chamber (Fine Science Tools, Vancouver, BC, Canada) perfused with a standard physiological solution containing (in mM) 124 NaCl, 26 NaHCO_3_, 3.5 KCl, 1 MgCl_2_, 1 CaCl_2_, and 10 D-glucose, equilibrated with 5% CO_2_ in 95% O_2_.

### 2.3. Electrophysiological recordings

We recorded the local field potential gradient (LFPg) with a 24 contact (distance between contacts: 150 μm) laminar microelectrode (Ulbert et al., 2001), and a custom-made voltage gradient amplifier of pass-band 0.01–10 kHz. Signals were digitized with a 32 channel, 16-bit resolution analog-to-digital converter (National Instruments, Austin, TX, USA) at 20 kHz sampling rate, recorded with a home written routine in LabView8.6 (National Instruments, Austin, TX, USA, RRID:SCR_014325). The linear 24 channel microelectrode was placed perpendicular to the pial surface, and slices were mapped from one end to the other at every 300–400 μm. Usually channels 1–8 were in the supragranular, channels 9–13 in the granular and channels 14–23 were in the infragranular layers. Channel positions were determined according to the thickness of the neocortex of the given patient and corrected if necessary.

### 2.4. Pharmacology

The AMPA (α-amino-3-hydroxy-5-methyl-4-isoxazolepropionic acid) and kainate types of GluRs were blocked by adding 2,3-dihydroxy-6-nitro-7-sulfamoyl-benzo(f)quinoxaline (NBQX) at a concentration of 5 μM, while NMDA (N-methyl-D-aspartate)-type receptors were blocked by adding DL-2-amino-5-phosphonovaleric acid (APV) at a concentration of 50 μM. These substances were procured from Tocris Bioscience (Izinta Kft, Budapest, Hungary). First, we recorded a control period (usually 5–10 min long), then applied the APV, followed by the joint application of APV and NBQX and a washout period. Continuous recordings were made from one spot on each slice comprising all phases of the pharmacological examination.

### 2.5. Data analysis

Spontaneous population activity detection, LFPg and multiunit activity (MUA) were analyzed with the Neuroscan Edit4.5 program (Compumedics Neuroscan, Charlotte, NC, USA), as described in our previous study. The LFPg peaks of the SPAs were taken as time zero for further event-related analyses. The location of SPAs – supragranular, granular, infragranular – was determined in each case. Baseline correction (−150 to −50 ms) was applied to averaged LFPg and MUA (Tóth et al., 2018).

For clustering we chose recordings with pharmacological applications, as we wished to examine the behavior of the human neocortical neurons in relation to the excitatory system. We clustered neurons in recordings from three slices from three non-epileptic patients and from four slices from three epileptic patients. Recordings were rendered compatible with the SpikeInterface framework (v. 0.97.0), aided by the MNE package (v. 1.2.2). Previously identified electrographic artifacts were imported and removed from the recordings, and a linear probe with 23 electrodes was used to set the characteristic probe scheme. At this step, SPA events (*n* = 5,589) were also assigned to the recording objects. Filtering thresholds between 300 and 6,000 Hz were set to maximize signal-to-noise ratios. Setting apart 300-s-long epochs for each pharmacological condition (control, APV, NBQX, and washout) did also take place, rejecting intervals considered transitionary between two states. Epochs from the same slices were handled together with the tridesclous spike sorting algorithm (v. 1.6.6.1), that found units above a 0.7 threshold, and the resulting spike trains were saved in a.h5 file. Waveforms, then template metrics, spike times and amplitudes, principal components, auto- and crosscorrelograms, inter-spike interval (ISI) histograms, unit locations and noise levels of each unit were computed and saved as CSV files. Waveform data was also exported to a Phy format, in order to alleviate manual curation. Burstiness index and the coefficient of variation (CV) of the ISIs were calculated and printed as a measure of the activity of the recorded units. All codes were written in Python programming language (v. 3.10).

For the classification of the clustered cells, we used our criteria described previously (Hofer et al., 2022). Briefly, based on the action potential (AP) waveform, we distinguished excitatory principal cells (PCs) and INs. If the AP width at half of its maximal LFPg amplitude (halfmax) was larger than 0.4 ms, the cell was considered to be PC, and IN if it was less than 0.2 ms. Discharge dynamics differentiated PC types as follows. A high peak at 3–10 ms followed by a fast exponential decay on the autocorrelogram was characteristic to intrinsically bursting PCs (IB-PCs). If the peak was lacking but there was sustained firing, or the peak was >10 ms, the cell was considered to be a regular spiking PC (RS-PC). The remaining cells with halfmax amplitude of >0.4 ms were categorized as PCs with unclear firing (UF-PC). A slow rise together with a slow decay identified INs. Cells with AP widths between 0.2 and 0.4 ms and non-characteristic autocorrelogram were defined as unclassified cells (UCs).

To examine how individual cellular activities could possibly contribute to SPAs, the following analysis was performed. We calculated the firing rate of each cell in two time windows: during the baseline (see above, −150 to −50 ms relative to the LFPg peak of the SPA) and peri-event (−50 to +50 ms) relative to time zero. We compared the firing rates in these two windows using non-parametric test and classified the cells into three categories: increased, decreased, or unchanged. A cell was considered increased (decreased) if its firing rate in the peri-event window was significantly higher (lower) than during the baseline window and the relative change was at least 50%. A cell was considered unchanged if there was no significant difference or the relative change was less than 50%. Additionally, Granger causality values between single unit activities and SPAs were calculated to infer causal relationships based on activity patterns. Granger causality can measure whether the past activity of one cell can help predict the future SPA better than the previous SPA alone.

The firing rate and the burstiness index were calculated as measures of activity of the units detected. The first metric constituted of the direct proportion between the number of single unit activities per epoch (expressed in number/second, Hz). Burstiness values were deducted from ISIs, which were computed from the spike train of each unit. Specifically, we identified a burst as a group of at least 3 spikes that occur within a time window of 20 ms. We marked the first and last spikes of each burst with burst start and end indices, respectively. The burstiness index was calculated as the number of spikes found within bursts, compared to overall spike count. This index provided a quantitative measure of how spikes were distributed across the recordings, with higher values indicating a greater degree of burstiness. The CV of the ISIs was also calculated as an optional measure of the unit activity.

Intrinsic relationships between single clusters were evaluated through their firing pattern crosscorrelograms. Each crosscorrelogram investigated single unit co-occurrences within a time window of −40 and +40 ms; taking into account 1 ms-long bins. A reliable relationship between two clusters was suspected if the maximum of at least one bin in the histogram exceeded the mean + 2 SDs of the crosscorrelogram baseline. To exclude false positive relationships due to low spike numbers, the following criteria was also considered: at least 20 spike counts should occur in the bin containing the maximum number of spikes. In cases where histogram peaks appeared at −3 and −1 ms range, a monosynaptic relation between two cells was declared, whereas peaks standing more than 3 ms behind time 0 were considered as polysynaptic connections.

### 2.6. Histological analysis

#### 2.6.1. Immunohistochemistry

Immunohistochemical procedures were used to verify the laminar structure and the possible tumor infiltration or signs of dysgenesis. Either tissue blocks or neocortical slices following electrophysiological recording were fixed and processed as described earlier (Wittner et al., 2009). Briefly, tissue samples were fixed with a fixative containing 4% paraformaldehyde and 15% picric acid and cut into 60 μm-thick sections with the vibratome described above. Neuronal cell bodies were stained with NeuN antibody (1:2,000, EMD Millipore, Billerica, MA, USA, RRID:AB_2298772), and the astroglial cells were stained with glial fibrillary acidic protein antibody (1:2,000, EMD Millipore, Billerica, MA, USA, RRID:AB_94844). Neocortical pyramidal cells were identified by the non-phosphorylated neurofilament protein SMI-32 antibody (1:4,000, Biolegend, San Diego, CA, USA, RRID:AB_2564642). All antibodies were mouse monoclonal antibodies. Their specificity was tested by the manufacturer. Visualization of the immunostained element was performed with the immunoperoxidase method, using biotinylated secondary antibody, avidin-biotin peroxidase complex and 3′3-diaminobenzidine, as the chromogene (Wittner et al., 2009). Sections were osmicated (20 min, 0.5% OsO_4_), dehydrated in ethanol and mounted in Durcupan (ACM; Fluka, Buchs, Switzerland).

#### 2.6.2. Cell counting

The density of SMI-32-stained cell bodies was determined in two non-epileptic and two epileptic samples. Non-overlapping images acquired with light microscopy using the 5× magnification objective were taken from all layers of the neocortex, to avoid sampling of the same cells. The number of the analyzed field of views varied between 12 and 32. The cell counting was made with a custom code in Python, that employed the OpenCV library (v. 4.7.0) to preprocess and analyze microscopic images. Beforehand, net tissue areas of the images were calculated in the ImageJ program (v. 1.53j), and multiplied by 60 μm (thickness of the section) to assess the volume. Images were split into separate RGB channels, where the ones with the highest contrast were selected. These files were loaded into the home-written routine and resized for easier handling. Images went under fastNlMeansDenoising algorithm to achieve noise reduction, and were applied adaptive thresholding for image segmentation. Next, Gaussian blur was used to further reduce noise, then morphological operations, such as opening and dilation were applied to remove small objects and fill gaps. Finally, a Canny edge detection method extracted edges and outlines for objects sizing between ±2 SDs were drawn and numbered as cell contours. Randomly chosen tissue snippets were also supposed to manual cell counting, in order to validate accuracy of the automated cell counting program.

#### 2.6.3. Electron microscopic analysis

The length of the synaptic active zones of excitatory axon terminals was examined in regions which generated SPA and in neighboring regions that did not show SPA. The following criteria were taken into consideration when choosing the samples for this quantitative electron microscopic analysis: the site that generated SPA and the site that did not generate SPA should be part of the same slice. The NeuN-positive section of the given slice should contain the whole length of the slice and the entire width including all neocortical layers. After light microscopic examination, areas of interest were re-embedded and sectioned for electron microscopy with a Leica EM UC6 ultramicrotome (Leica GmBH, Wetzlar, Germany, RRID:SCR_020226). Ultrathin serial sections were collected on Formvar-coated single slot grids, stained with lead citrate, and examined with a Hitachi 7100 electron microscope (Hitachi, Tokyo, Japan). The same electron microscopic samples were used, as the ones for the analysis of synaptic connectivity performed in our previous study (Tóth et al., 2018). SPA occurred in the supragranular layers in all selected slices. Two blocks were re-embedded from the supragranular layers (upper layer III) of each slice, one from the spot where SPA was recorded and the other from an area where SPA was not generated. Photos were systematically taken at 20,000× magnification, from one side to the other side of the block without overlapping areas, to avoid multiple sampling of the same synapse. The number of photos analyzed per patient varied from 16 to 31. The length of the synaptic zone of the excitatory (asymmetrical) synapses was measured with the ImageJ program (Schneider et al., 2012). The target elements of the asymmetrical synapses were determined based on the following anatomical features. Spines were usually small structures with no organelles, whereas dendrites were large, and contained mitochondria and/or microtubules. Large target elements without organelles or targets cut at the side of the photo were classified as unidentified elements.

### 2.7. Statistical analysis

We conducted statistical analysis relying on the GraphPad Prism software (v. 8.0.1) and SciPy (v. 1.10.1). Firstly, to ensure the validity of our control group, we performed a statistical analysis to test whether age had a non-negligible effect on the results displayed in our study. We used a multiple regression model with age and the condition of epilepsy as independent variables and perforated synapses as the dependent variable. Next, the normality of all data was tested by the Agostino–Pearson test. If the data was normally distributed, we performed a two-way ANOVA to analyze the effects of two independent variables on a dependent variable. For data with a single independent variable, we used either a one-way ANOVA or Kruskal–Wallis test, depending on whether the assumption of normality was met. Specifically, the Kruskal–Wallis test was used in the lack of Gaussian data distribution, with Dunn’s follow-up test to perform multiple comparisons parallelly with statistical hypothesis testing. Furthermore, when comparing two groups and the data was not normally distributed, a Mann–Whitney test was used instead of an unpaired *t*-test. As a substitute for the paired *t*-test, Wilcoxon’s matched-pairs signed rank test was used for data not passing normality criteria. Additionally, in cases where we had categorical data, we used a Chi-square test to assess the association between two or more categorical variables. The choice of statistical analysis method was based on the nature of the data and the research question being asked, and data following Gaussian distribution were described as mean ± SEM. In addition, datasets not passing the normality tests are also characterized by their median and the first and third quartiles confidence interval of the median.

## 3. Results

### 3.1. Spontaneous population activity

**FIGURE 1:**
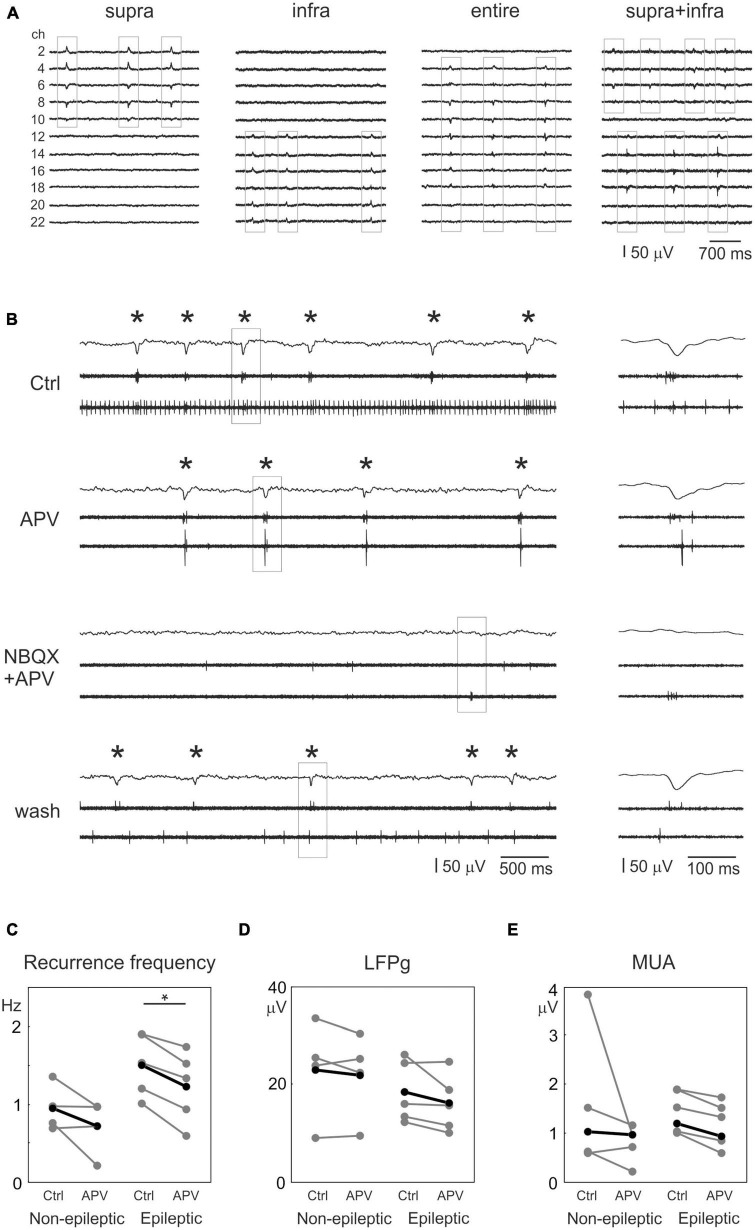
Role of glutamate receptors in the generation of spontaneous synchronous population activity. **(A)** Recordings of spontaneous population activity (SPA) arising from different layers of a human neocortical slices, **(B)** SPAs and simultaneous single unit activities of a neocortical slice derived from a non-epileptic patient, during the consequent application of physiological solution – control, the NMDA receptor antagonist APV, APV + NBQX (NMDA and AMPA/kainate receptor antagonists), and finally physiological solution – washout. Local field potential gradients (LFPg), i.e., recordings filtered between 1 and 30 Hz are presented on the top traces, while lower extracts display the action potentials of single cells after high-pass filtering at 500 Hz. Asterisks indicate the occurrence of SPAs. Note the reduced presence of SPAs during APV bathing solution; while SPAs, but not all action potentials are abolished when NBQX is also applied. SPAs return after washing out of the pharmacological agents. Framed sections are displayed in detail on the right. Statistical comparisons between control conditions and application of APV for various parameters: **(C)** SPA recurrence frequency, **(D)** LFPg amplitudes and **(E)** multiunit activity (MUA) amplitudes. Note that the recurrence frequency of SPA has significantly decreased in epileptic tissue upon application of APV (*p < 0.05).

Spontaneous population activity (SPA) was generated in 20/40 and in 20/35 slices derived from non-epileptic and epileptic patients (Figure 1A), respectively (see also Tóth et al., 2018; Kandrács et al., 2019; Hofer et al., 2022). SPAs consisted of recurring population bursts consisting of local field potential transients with increased high frequency oscillations and cell firing. In our previous study, six SPA types were differentiated based on their location and extension within the neocortex: supragranular, supragranular + granular, granular, granular + infragranular, infragranular, and one type invaded the entire width of the neocortex (Tóth et al., 2018). One population burst will be called an SPA event, whereas the totality of the recurring population events at one location and within one recording will be referred to as SPA. Single SPA (i.e., one type of SPA) emerged in 15 and 13 slices from non-epileptic and epileptic samples, respectively, whereas in the remaining slices (5 and 7) multiple SPAs were observed (Figure 1A). Both simultaneous multiple SPAs in one recording site and multiple spots of SPAs in one slice were recorded. Altogether, 30 and 28 SPAs were detected in non-epileptic and epileptic tissue, respectively. Most of the SPAs (22 in non-epileptic, 23 in epileptic samples) emerged in the supragranular layers, the remaining SPAs were detected in the infragranular layers, and in one case we observed an SPA invading the entire width of the neocortex (in a slice derived from a non-epileptic patient, see the different types on Figure 1A).

Next, we analyzed the recurrence frequency, the LFPg and the multiple unit activity (MUA) amplitudes of the SPAs (Table 2 and Figure 1). The number of SPA events in a given recording varied between 74 and 1,744, with a median (first and third quartile) of 323.5 [193.5–627.5] in non-epileptic (*n* = 26 SPAs) and of 422.5 [172–610.25] in epileptic (*n* = 28 SPAs) tissue. The recurrence frequency of the SPA events was similar in non-epileptic and epileptic tissue: 0.97 [0.71–1.46] Hz and 1.02 [0.80–1.63] Hz, respectively. The LFPg amplitudes were also not different, such as the MUA amplitudes: LFPg amplitude in non-epileptic: 20.6 [12.8–28.8], in epileptic: 24.1 [15.9–30.3] μV, MUA amplitude in non-epileptic: 0.87 [0.57–1.82] μV, epileptic: 0.91 [0.45–1.28] μV (*p* > 0.05, Mann–Whitney U test, for all parameters).

**TABLE 2:** Network properties of SPAs.

	Number of SPA	Recurrence frequency	LFPg amplitude	MUA amplitude
Non-epileptic	*n* = 26	1.16 ± 0.14 0.97 [0.71–1.46]	22.14 ± 2.24 20.60 [12.82–28.82]	1.45 ± 0.28 0.87 [0.57–1.82]
Epileptic	*n* = 28	1.18 ± 0.13 1.02 [0.80–1.63]	27.04 ± 3.28 24.10 [15.86–30.30]	1.14 ± 0.19 0.91 [0.45–1.28]

Data are shown in mean ± standard error of the mean (SEM) and median (first to third quartiles). Epileptic is not different from non-epileptic (Mann–Whitney U test, *p* > 0.05).

### 3.2. Blockade of the glutamate signaling

Antagonizing AMPA/kainate types of GluRs abolished the generation of epileptiform synchronous population bursts in human subicular (Cohen et al., 2002) and neocortical (Köhling et al., 1998) slices derived from epileptic patients, whereas blockade of NMDA type GluRs had no considerable effect on these population bursts (Köhling et al., 1998). We wished to examine the role of the glutamatergic signaling in the generation of SPAs in both epileptic and non-epileptic human neocortical slices (see also Tóth et al., 2018). Therefore, we blocked the glutamate signaling by applying the NMDA GluR antagonist APV (50 μM) and further completed the bathing solution with the AMPA/kainate GluR antagonist NBQX (5 μM) in *n* = 3 slices from three non-epileptic patients and in *n* = 5 slices from four epileptic patients. Single SPAs were generated in all but one slice during the application of physiological bath. Two types of SPAs emerged in one slice derived from non-epileptic tissue, one in the supragranular, one in the infragranular layers in physiological solution (Figure 1A). The blockade of the NMDA receptors significantly reduced the recurrence frequency of SPAs in epileptic (*n* = 5 SPAs) but not in non-epileptic tissue (*n* = 4 SPAs, Table 3 and Figures 1B, C, paired t-test, *p* < 0.05). All the other changes were not significantly different (Table 3 and Figures 1D, E). Further antagonizing the AMPA/kainate receptors reversibly blocked the emergence of SPAs in both patient groups. Application of APV in the slice with two simultaneously occurring SPA did not induce significant changes, while adding NBQX blocked the appearance of both SPAs. After a long washout period both SPAs reappeared, however, the recurrence frequency of the SPAs did not reach the value observed before applying GluR antagonists. Washing out NBQX usually took a very long time, and in two slices (one from epileptic, one from non-epileptic sample) the washout was not successful even after 40 min. We have to note that otherwise, SPA is typically an unchanging, recurring population activity which can be maintained for more than 3 h in our recording system, with a stable recurring frequency, LFPg and MUA amplitudes (Tóth et al., 2021).

**TABLE 3:** Effect of APV application on the properties of SPAs.

	Number of SPA		Average number of SPA events	Recurrence frequency	LFPg amplitude	MUA amplitude
Non-epileptic	*n* = 4	Control	448 ± 93	0.95 ± 0.15 0.87 [0.74–1.07]	23.15 ± 5.16 24.86 [20.30–24.60]	1.53 ± 0.79 0.83 [0.62–1.74]
		APV	227 ± 70	0.72 ± 0.18 0.84 [0.59–1.53]	22.05 ± 4.50 24.00 [19.31–26.74]	1.17 ± 0.53 0.71 [0.57–1.31]
Epileptic	*n* = 5	Control	439 ± 186	1.51 ± 0.18 1.54 [1.21–1.91]	18.57 ± 2.89 16.12 [13.48–24.60]	1.04 ± 0.36 0.80 [0.70–1.07]
		APV	313 ± 81	1.23 ± 0.21* 1.34 [0.94–1.53]	16.28 ± 2.65 15.79 [11.63–19.01]	0.85 ± 0.22 0.78 [0.74–0.83]

Data are presented both in mean ± SEM and median (first to third quartiles).

*Significantly different from control, *p* < 0.05, paired *t*-test.

### 3.3. Cell clustering

We aimed to investigate the behavior of human neocortical neurons in relation to excitatory signaling. Therefore, single cells were clustered from recordings obtained during control conditions and blockade of NMDA and AMPA/kainate GluRs. In three and four recordings derived from non-epileptic and epileptic samples, we clustered 30 and 34 neurons, respectively.

**FIGURE 2:**
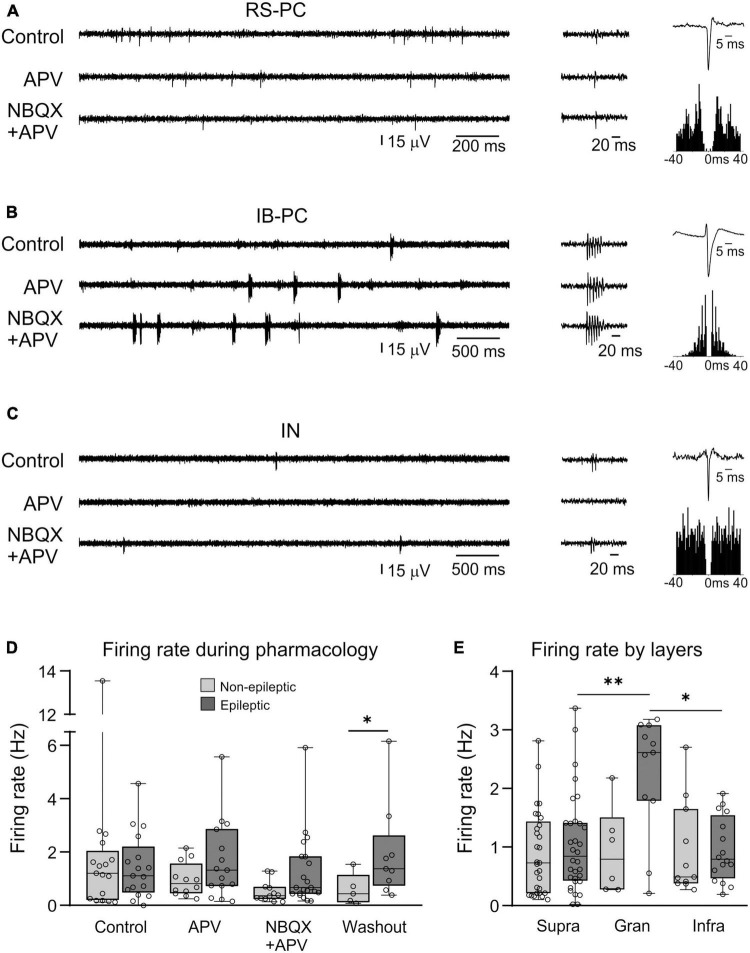
Single unit firing patterns attributed to various cell types during the pharmacological experiments. Left panels show the firing patterns of **(A)** regularly spiking principal cells (RS-PCs), **(B)** intrinsically bursting principal cells (IB-PCs), and **(C)** interneurons (IN). Middle panels show magnified single units, while right panels present average action potential waveforms and autocorrelograms of the respective cell. **(D)** Firing rate changes of all cells (non-epileptic vs. epileptic) during the pharmacological investigations. **(E)** Firing rates attributed to cells located in the supragranular, granular, and infragranular layers of the neocortex (*p < 0.05; **p < 0.01).

In the non-epileptic tissue, we found 20 excitatory PCs (60%), three inhibitory cells (IN, 10%), and 10 UCs (30%). In epileptic tissue, 16 PCs (47%), 10 INs (29%), and 8 UCs (24%) were detected. In non-epileptic tissue four IB-PCs, seven RS-PCs, and seven UF-PCs were distinguished. The ratio of the different PC types was similar in epileptic tissue, four IB-PCs, five RS-PCs, and seven UF-PCs were clustered (Table 4 and Figures 2A–C).

**TABLE 4:** Numbers and percentages of different cell types among the clustered neurons.

		*n*	All PCs (%)	RS-PC (%)	IB-PC (%)	UF-PC (%)	IN (%)	UC (%)
Non-epileptic	Total	30	18 (60)	7 (23.3)	4 (13.3)	7 (23.3)	3 (10)	9 (30)
	Control	20	12 (60)	5 (25)	2 (10)	5 (25)	1 (5)	7 (35)
	APV	14	11 (78.6)	4 (28.6)	4 (28.6)	3 (21.4)	1 (7.1)	2 (14.3)
	NBQX + APV	14	11 (78.6)	5 (35.7)	2 (14.3)	4 (28.6)	1 (7.1)	2 (14.3)
	Washout	13	9 (69.2)	4 (30.8)	2 (15.4)	3 (23.1)	0 (0)	4 (30.8)
Epileptic	Total	34	16 (47.1)	5 (14.7)	4 (11.8)	7 (20.6)	10 (29.4)	8 (23.5)
	Control	21	11 (52.4)	4 (19.1)	3 (14.3)	4 (19.1)	5 (23.8)	5 (23.8)
	APV	18	12 (66.7)	5 (27.8)	3 (16.7)	4 (22.2)	3 (16.7)	3 (16.7)
	NBQX + APV	22	11 (50)	4 (18.2)	4 (18.2)	3 (13.6)	7 (31.8)	4 (18.2)
	Washout	16	8 (50)	3 (18.8)	3 (18.8)	2 (12.5)	4 (25)	4 (25)

Percentages of RS-PC, IB-PC, and UF-PC are relative to the total number of neurons.

When analyzing single cells depending on their location in cortical depth, we did not identify significant differences between numbers or proportions of neurons located in supragranular (channels 1–8), granular (channels 9–13), or infragranular (channels 14–23) layers, whether they originated from non-epileptic or epileptic tissue (*p* = 0.51, Chi-square test).

### 3.4. Neuronal firing rate

As we performed continuous recording when applying the pharmacological agents, we could follow the single cells in physiological solution and during drug applications. All different patterns of neuronal discharge were observed: cells firing in physiological bath could be either continue or stop firing during drug application, as well as silent (and thus non-detected) cells in control conditions could start discharging during any of the drug application steps. As washout usually took a very long time, the recording was not continuous in several cases, and therefore, the same cells could not always be followed. Table 4 shows the numbers of the active (clustered) neurons in different pharmacological conditions, as well as their excitatory or inhibitory nature.

**FIGURE 3:**
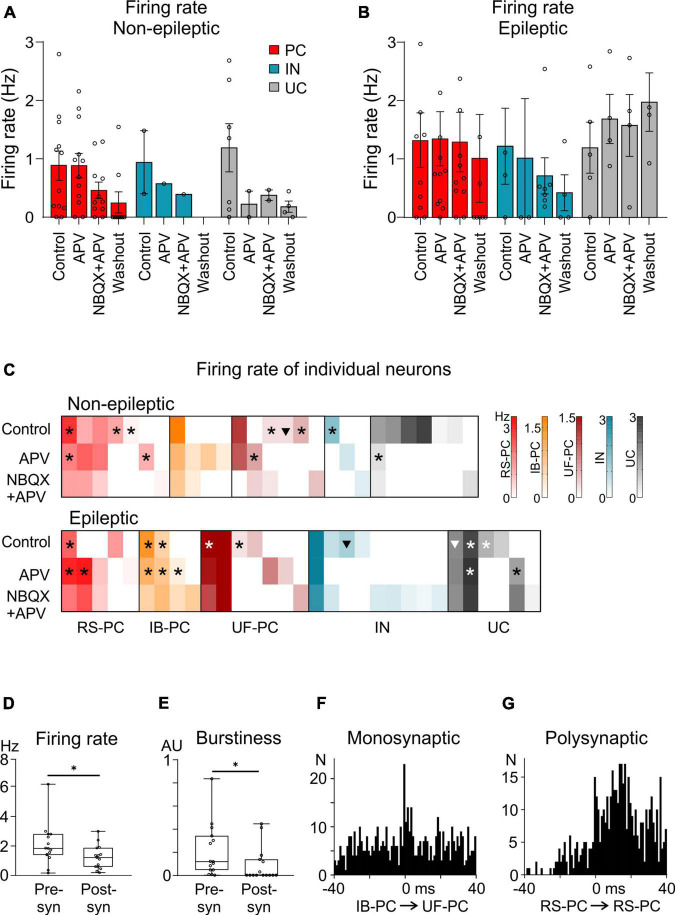
Investigation of the firing rates in different conditions. Cellular subtypes (PC, IN, and UC) and their firing rates with regards to the different pharmacological conditions, in non-epileptic **(A)** and epileptic tissue **(B)**. Firing rates in epileptic tissue were not significantly higher than in non-epileptic samples. **(C)** Heatmaps displaying individual cells’ firing rates along the different pharmacological conditions (Control, APV, NBQX + APV). Color codes express cell class dependence (warm colors for PCs with different firing patterns, blue for IN, gray for unclassified cells, UCs), whereas color gradients show firing rate values (in Hz). Symbols inside the boxes indicate that the cell significantly changed its firing rate during SPA: asterisk stands for increased, triangle for decreased firing rate during population activity. Subplots **(D–F)** are based on the crosscorrelograms of reliable cell connections, evaluating presynaptic and postsynaptic cell members’ **(D)** firing rate and **(E)** burstiness index differences, both significantly different (*p < 0.05). **(F)** Crosscorrelogram of a cell pair in a monosynaptic relationship. Note that the IB-PC can evoke burst firing in the unknown firing PC (UF-PC). **(G)** Polysynaptic connection between two RS-PCs, detected based on this crosscorrelogram.

Only part of the cells fired spontaneously during control, then APV, then NBQX + APV conditions: five cells (16.7%) in non-epileptic and eight cells (23.55%) in epileptic tissue (Figure 3C). Out of these, most of them were PCs (four cells in non-epileptic and five in epileptic tissue), and only one was an IN (in epileptic tissue). Numerous neurons which were spontaneously active in control conditions stopped firing during the application of APV (11 in non-epileptic, 8 in epileptic samples), and only two of them (1 non-epileptic, 1 epileptic) started discharging again during NBQX application (see Figure 3C). On the other hand, several neurons which were silent in physiological bathing solution started to fire when APV was applied (six in non-epileptic, seven in epileptic tissue). Out of these cells, two in non-epileptic, three in epileptic tissue kept firing during the application of NBQX as well, whereas the remaining neurons stopped discharging. Four cells in non-epileptic and eight in epileptic samples started firing only in NBQX containing bathing solution, interestingly, most of these were INs (one IN in non-epileptic, five INs in epileptic samples). In summary, all different kinds of firing behaviors were observed: continuous firing during physiological bath and blocked glutamate signaling, or discharge only during one or more pharmacological steps. Numerous PCs including both RS-PCs and IB-PCs discharged during the three phases (control, APV, and NBQX), whereas a high number of INs started to fire only in NBQX bath in epileptic tissue.

We examined the firing rate of the clustered cells during the pharmacological bath application (Table 5 and Figure 3). Note that the number of neurons per cell type group and pharmacological condition is quite low (0–7), therefore the following results might be distorted by the incidental sampling. On average, GluR antagonists did not elicit significant changes in neuronal firing rate, neither in epileptic, nor in non-epileptic tissue. However, the firing rate of all cells in non-epileptic tissue (*n* = 20 cells in control) gradually decreased from 1.01 ± 0.21 Hz during the application of GluR antagonists (*n* = 14 and 14 cells in APV and in APV + NBQX solution) and washout (*n* = 13 cells), although this change was not significantly different from the control (Figure 3D). In epileptic tissue the firing rate of all cells was 1.17 ± 0.27 Hz in physiological conditions (*n* = 21 cells) similar to non-epileptic) and remained above 1 Hz during the pharmacological experiments (*n* = 18 and 22 cells in APV and in NBQX solution, respectively) and washout (*n* = 16 cells, Figure 3D). The firing rate of PCs (*n* = 18 and 16 PCs in non-epileptic and in epileptic samples, respectively) and INs (*n* = 3 and 10 INs, in non-epileptic and epileptic tissue, respectively) was also similar in both patient groups, and did not change during drug application. Neither could any significant changes between RS-PC (*n* = 7 in non-epileptic and *n* = 5 in epileptic tissue) and IB-PC (*n* = 4 in non-epileptic, *n* = 4 in epileptic) firing rates be pointed out.

**TABLE 5:** Firing rates and burstiness of distinct cell types depending on the pharmacological conditions.

		Control		APV		NBQX		Washout	
		**Firing rate**	**Burstiness**	**Firing rate**	**Burstiness**	**Firing rate**	**Burstiness**	**Firing rate**	**Burstiness**
Non-epileptic	Total	1.01 ± 0.21 0.98 [0.14–1.63]	0.04 ± 0.02 0.004 [0–0.04]	0.79 ± 0.2 0.8 [0–1.86]	0.21 ± 0.10 0.005 [0–0.6]	0.47 ± 0.11 0.33 [0.14–0.7]	0.2 ± 0.12 0 [0–0.59]	0.21 ± 0.11 0 [0–0.4]	0 ± 0
	PC	0.85 ± 0.27 0.45 [0–1.72]	0.08 ± 0.04 0.02 [0–0.17]	0.93 ± 0.24 0.82 [0–1.86]	0.26 ± 0.12 0.01 [0–0.64]	0.5 ± 0.14 0.31 [0.14–1.26]	0.26 ± 0.16 0.02 [0–0.77]	0.25 ± 0.18 0 [0–0.72]	0 ± 0
	IN	1.48 ± 0 1.48	0.006 ± 0 0.006	0.57 ± 0 0.57	0.01 ± 0 0.0116	0.39 ± 0 0.39	0 ± 0	N/A	N/A
	UC	1.18 ± 0.41* 1.35 [0–2.68]	0.009 ± 0.008 0 [0–0.01]	0.22 ± 0.22 0.22 [0–0.44]	0 ± 0	0.28 ± 0.18 0.37 [0.28–0.46]	0 ± 0	0.17 ± 0.09 0.13 [0–0.44]	0 ± 0
Epileptic	Total	1.17 ± 0.27 0.72 [0.16–1.64]	0.18 ± 0.06 0.08 [0.001–0.48]	1.40 ± 0.34 0.91 [0.23–2.14]	0.05 ± 0.02 0 [0–0.09]	1.19 ± 0.28 0.63 [0.44–1.83]	0.07 ± 0.03 0 [0–0.16]	1.11 ± 0.41 0.48 [0–1.76]	0.1 ± 0.07 0.008 [0–0.14]
	PC	1.20 ± 0.44 0.6 [0–2.98]	0.32 ± 0.07 0.37 [0.17–0.43]	1.35 ± 0.46 0.77 [0.23–2.14]	0.09 ± 0.04 0.04 [0–0.22]	1.36 ± 0.49 0.67 [0.44–2.38]	0.18 ± 0.06 0.22 [0–0.3]	1.01 ± 0.75 0 [0–6.16]	0.003 ± 0.003 0 [0–0.004]
	IN	1.06 ± 0.53 0.72 [0–2.98]	0.23 ± 0.13^#^ 0.13 [0.04–0.52]	1.02 ± 1.02 0 [0–3.06]	0 ± 0	0.71 ± 0.31 0.49 [0.18–2.55]	0 ± 0	0.42 ± 0.3 0.19 [0–1.31]	0.074 ± 0
	UC	1.2 ± 0.43 1.08 [0–2.59]	0.001 ± 0.001 0 [0–0.005]	1.96 ± 0.45 1.71 [1.32–2.85]	0.001 ± 0.001 0 [0–0.005]	1.58 ± 0.53 1.7 [0.17–2.74]	0.001 ± 0.001 0 [0–0.004]	1.98 ± 0.5 1.83 [0.93–3.34]	0.19 ± 0.13^&^ 0.08 [0.001–0.4]

Significances: *p* < 0.05, two-way ANOVA multiple comparisons *post-hoc* test.

*Firing rate of UCs in non-epileptic tissue: control differs from all other conditions.

^#^Burstiness of INs in epileptic tissue: control differs from all other conditions.

^&^Burstiness of UCs in epileptic tissue: washout differs from all other conditions.

Next, we examined the firing rate of single cells during the different pharmacological applications. Single cells with firing through all the three phases (control, APV, and NBQX + APV), showed either increasing, decreasing, or stable firing rates (Figure 3C). In non-epileptic tissue, most cells (23/27, 85.2%) displayed a lower firing rate when we applied NBQX + APV bath, than before. The remaining four cells (two PCs, one IN, and one UC) started to discharge only in the solution containing NBQX + APV. In epileptic tissue, only 19/31 cells (61.3%) showed a lower firing rate in NBQX + APV bath. The eight cells (two PCs, five INs, and one UCs) which started firing only in APV + NBQX solution displayed a low firing rate (0.4 ± 0.06 Hz).

When we examined firing rates in relation to cellular location, we observed that granular layer-based cells had significantly higher firing rate values in epileptic tissue (*n* = 7 cells), compared to the non-epileptic ones (epileptic_granular: 2.21 ± 0.31; 2.59 [1.76–3.05] vs. non-epileptic_granular: 0.91 ± 0.3; 0.77 [0.25–1.48], Figure 3B). It must be noted that out of the seven cells detected on granular layer channels, three were identified as interneurons, whereas no IB-PCs were found.

### 3.5. Burstiness

Based on their firing properties (by examining the autocorrelogram), we classified the PCs as regular spiking (RS-PCs) or intrinsically bursting (IB-PCs). Besides this, we determined the burstiness (see section “2. Materials and methods”) of all neurons and examined the possible effects of the pharmacological manipulations on the bursting properties. As in case of the firing rate, all the following data should be viewed with precaution, since the neuronal numbers are low. Contrary to our previous study (Hofer et al., 2022), the burstiness of all cells in physiological solution, as well as in all pharmacological conditions was higher in epileptic tissue than in non-epileptic tissue (0.18 ± 0.06; 0.08 [0.001–0.48] in epileptic vs. 0.04 ± 0.02; 0.004 [0–0.17] in non-epileptic, Dunn’s multiple comparison test, *p* = 0.015, Table 5).

When looking at the effects of GluR antagonists, the burstiness of INs in epileptic tissue became lower when blocking GluRs, compared to control conditions, whereas UCs showed a very high burstiness during washout, compared to all previous conditions (for values see Table 5). Although we could not demonstrate any effect of the used pharmacological agents on the burstiness of the different PC types (RS-PCs and IB-PCs), IB-PCs in NBQX + APV bath showed a significantly lower burstiness in epileptic (0.27 ± 0.04; 0.27 [0.20–0.35]) compared to non-epileptic (0.78 ± 0.007; 0.78 [0.77–0.789]) tissue (Kruskal–Wallis test with Dunn’s follow-up test, *p* < 0.01).

### 3.6. Single unit firing properties during SPA

We intended to get an insight from the involvement of the clustered cells in the generation of the SPAs detected. Therefore, we investigated the firing rate change of the neurons relative to the SPAs (see section “2. Materials and methods”). For cells with increased firing rate, Granger causality calculations were performed, as an exploration of inherent causalities. The investigations also took place separately in physiological solutions and in the presence of APV (in NBQX + APV SPAs were blocked, therefore this analysis was not performed). In non-epileptic tissues we examined 29 neurons in the two different conditions (17 cells in control and 12 in APV). Six cells in control and 5 cells in APV solution exhibited increased firing (out of which 5/29 passed Granger causality criteria), 1 cell decreased its activity in control, whereas the remaining cells had unchanged firing rate during SPA.

In epileptic samples we analyzed 16 cells in control and 15 neurons in APV bath; 7 neurons in control and 7 in APV had increased (13/31 passing Granger causality test), 2 cells in control had decreased and all others had unchanged firing rate during SPA. The ratio of increased and decreased firing rates was not significantly different between the experimental groups (*p* = 0.19, Fisher’s exact test). Decreased single unit firing patterns along the SPAs could be observed in physiological, but not in APV bath. Cells investigated in the epileptic group (*n* = 31) showed a higher, but non-significant firing increase during SPAs, compared to the non-epileptic values (*n* = 26, 636 ± 175.8%, 111.1 [90.09–4,100] vs. 298.5 ± 79.42%, 120.2 [76.34–277.6]; *p* > 0.05, Mann–Whitney test). Neither could any significant difference between physiological solution – APV conditions been observed, regardless of the epileptic or non-epileptic groups (non-epi control: 499.5 ± 258.5%, 136.2 [89.47–1,091], non-epi APV: 364.4 ± 254.7%, 128.1 [76.78–758.6]; epi control: 976.1 ± 382.1%, 812 [83.07–1,521]; epi APV: 895 ± 480.2%, 425 [103.5–1,000]; *p* > 0.05, Kruskal–Wallis test). Analyzing distinct cell types revealed that IB-PCs recorded the epileptic group increased their firing rates significantly more than those from non-epileptic tissue (4,020 ± 2,185% vs. 89.16 ± 7.63%, *p* = 0.0025, Mann–Whitney test), but the in-group differences between physiological conditions and APV application were not substantially different. In total, 3/5 of the IB-PC did not elicit any single unit activity before the beginning of SPAs, thus rendering the firing change infinite. Although cells with increased firing during SPAs were not significantly changing values between physiological and APV conditions, epileptic tissue cells with unchanged firing during SPAs significantly reduced their firing along the application of APV (control: 106.2 ± 1.89%, 105 [102.4–110.6] vs. APV: 83.2 ± 6.6%, 84.3 [65–98.33]; *p* = 0.01, Mann–Whitney test).

### 3.7. Putative synaptic interactions

We computed crosscorrelograms to analyze temporal relationships between the firing activities attributed to neurons. Out of the 698 possible combinations, 15 crosscorrelograms passed the criteria of being either a monosynaptic (*n* = 10) or a polysynaptic (*n* = 5) relationship between two cells (see section “2. Materials and methods,” Figures 3F, G). Analyzing cell locations, we noted that 11 of these connections were detected on infragranular layer recording channels, whereas 3 cell pairs were identified on neighboring supragranular-granular channels. All investigated cell classes participated in these synaptic connections, but only one proved to be PC-IN connection. In 8 pairs at least one cell was an IB-PC, while the rest of the cells represented RS-PCs and UCs in several combinations. All connections involving IB-PCs derived from five neurons, and in six out of eight cases, the IB-PC formed the presynaptic member of the cell pair. The overrepresentation of IB-PCs was reliably detectable among the connections (5 out of the 8 total number of identified IB-PC cells, in comparison to the other cell types’ ratio of 9 out of a total of 56 cells, *p* = 0.04, Fisher’s exact test). Presynaptic members of the cell pairs did show significantly higher firing rates compared to the postsynaptic cells (Figure 2D, 2.06 ± 0.36; 1.83 [1.39–2.79] vs. 1.22 ± 0.2; 1.21 [0.6–1.86], *p* = 0.045), and burstiness indices did also reveal this feature (Figure 2E, 0.2 ± 0.05; 0.11 [0.04–0.33] vs. 0.08 ± 0.03; 0 [0–0.13], *p* = 0.04).

### 3.8. Anatomical examinations

**FIGURE 4:**
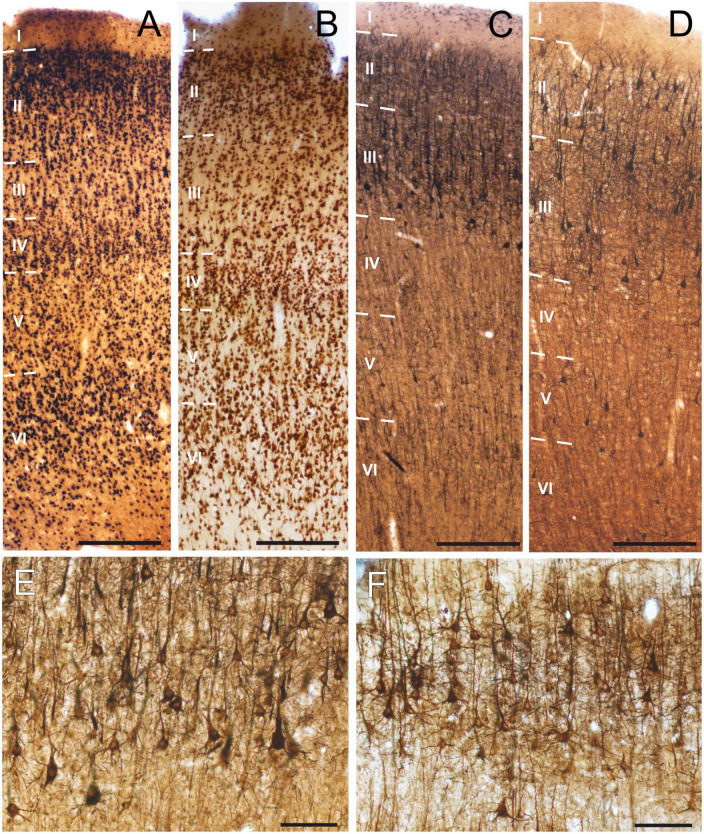
Histological analysis of non-epileptic and epileptic human neocortices. Neocortical tissue was stained against the neuronal marker NeuN, both in the **(A)** non-epileptic and **(B)** epileptic group. SMI-32 staining in non-epileptic and epileptic tissues highlighted projecting pyramidal cells either from the complete thickness of the neocortex **(C,D)** or focusing on layer 3 **(E,F)**. Note that while background intensity averages may vary between epileptic and non-epileptic tissue, there is no significant change in neuron density. All samples derived from the temporal lobe. Scale bars: **(A–D)** 500 μm, **(E,F)** 100 μm.

We verified the structure of the neocortex in sections stained against the neuronal marker NeuN in both non-epileptic (*n* = 6) and epileptic samples (*n* = 7). The six layers of the neocortex were visible in all cases (Figures 4A, B). The neocortex was infiltrated by the tumor in two non-epileptic cases: in one sample we saw slightly distorted pyramidal cells in a patch in layers 5/6, whereas in another sample several large round neurons were observed. The signs of the dysgenesis were visible in tissues derived from two epileptic patients: in one sample numerous neurons were visible in the white matter, whereas in the other one misplaced neurons and large balloon-like cells were observed. All the other samples (*n* = 4 tumor and *n* = 5 epileptic) were having normal structure.

To assess possible changes in the excitatory system in epilepsy, we examined the distribution of the neocortical pyramidal cells expressing non-phosphorylated neurofilament protein SMI-32 (Figures 4C–F). As described earlier (Del Rio and DeFelipe, 1994; Fauser et al., 2013), SMI-32-stained neurons were located mainly in layers III, V, and VI, with an inhomogeneous distribution. In several patches numerous SMI-32-positive cells were present, whereas certain areas were almost empty, and immunostaining contrast intensities were also considerably lower at these sites. Neuronal density decreases in the human epileptic neocortex compared to non-epileptic samples (Thom et al., 2005; Tóth et al., 2018). We wished to know whether this cell loss affects the SMI-32 pyramidal cell population. Therefore, we counted the number of neurons in eight sections derived from two non-epileptic patients and in three sections in two epileptic patients. Altogether, we counted 14,034 cells and calculated neuronal densities in every examined section. SMI-32-positive neuron densities were similar in the two patient groups: 51.74 ± 14.93 cells/100 μm^3^ in non-epileptic, and 46.35 ± 9.98 cells/100 μm^3^ in epileptic tissue (*p* = 0.46, unpaired *t*-test with Welch’s correction).

### 3.9. Size of the synaptic active zones

**FIGURE 5:**
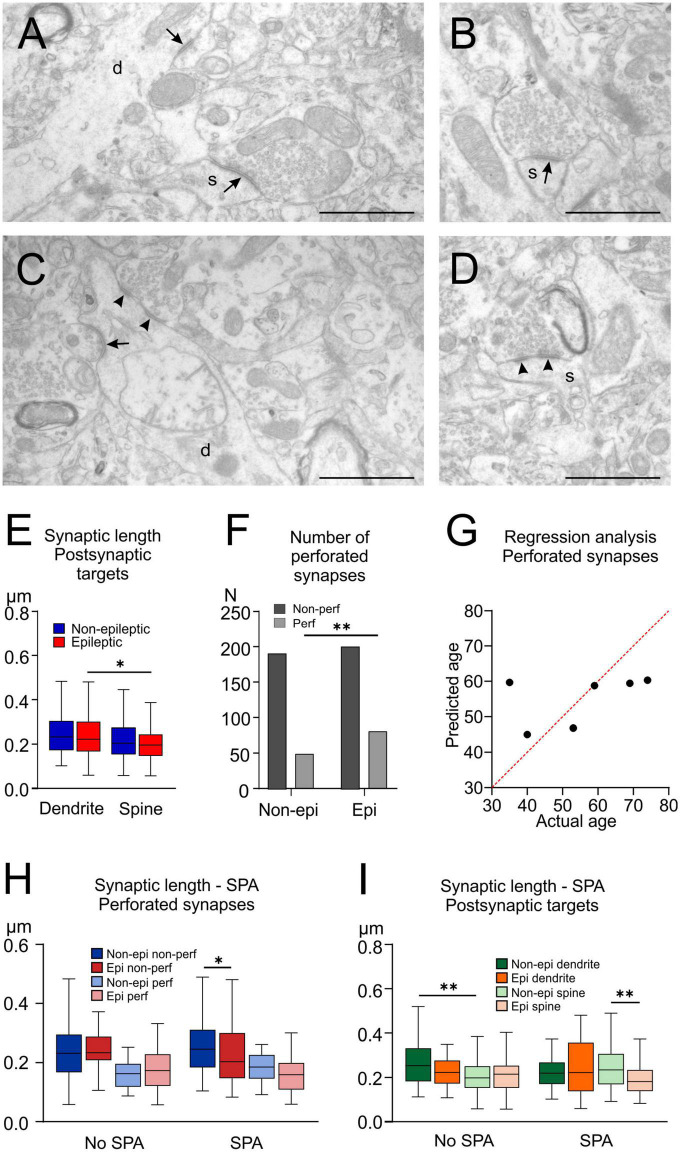
Analysis of excitatory synapses at electron microscopic level. **(A–D)** Electron micrographs reveal asymmetrical (excitatory) synaptic active zones terminating on dendrites (**A,C**, labeled with “d”) or spines (**A,B,D**, annotated with “s”), and having the synaptic active zone uninterrupted (arrows on **A–C**) or split, resulting in perforated synapses (**C,D**, marked with arrowheads). Low percentage (4.6% in non-epileptic and 2.5% in epileptic) of dendrites received multiple synapses (dendrite on **C**) at the examined single plane. Scale bars: 1 μm. **(E)** The active zone length of synapses terminating on spines was lower than that on dendrites in epileptic tissue (*p < 0.05). **(F)** The number of perforated synapses was higher in epileptic compared to non-epileptic tissue (**p < 0.01). **(G)** Multiple regression analysis displaying the actual and predicted age of the patients, from whom tissue samples were derived, calculated on the ratio of perforated synapses. This analysis meant to demonstrate that the discrepancy between epileptic and non-epileptic groups’ mean age could not significantly distort results based on histological investigations. **(H)** Considering non-perforated synapses in SPA-generating regions, epileptic tissue had shorter synaptic active zones, compared to non-epileptic ones (*p < 0.05). **(I)** The active zone length of synapses terminating on dendrites was higher than on spines in the non-epileptic tissue, in regions lacking SPA (No SPA), whereas in regions generating SPA length of synaptic active zones terminating on spines were lower in epileptic compared to non-epileptic samples (**p < 0.01).

In temporal lobe epilepsy, the epileptic synaptic reorganization affects both the excitatory (Rossini et al., 2021) and inhibitory systems (Wittner and Maglóczky, 2017) of the human hippocampus. In our previous study we showed that the density of excitatory synapses increases in human neocortical epilepsy, compared to non-epileptic tissue (Tóth et al., 2018). Although the density of inhibitory synapses remained similar in our samples (Tóth et al., 2018), we found that the size of the inhibitory synapses (i.e., the size of the active zones) of the parvalbumin-positive basket cells was enhanced in regions where SPAs emerged (Tóth et al., 2021). Usually, SPA emerged in a circumscribed area of the slice, while SPA could not be detected in other parts of the same slice (Tóth et al., 2018). We hypothesized that the microanatomical features – and mainly those of the excitatory system – might explain why several microcircuits are prone to generate synchronies while others are not. Therefore, in the present study we examined the size of the excitatory axon terminals in relation to epilepsy and to SPA generation. To see possible changes in the glutamatergic system, we measured the length of the synaptic active zones of asymmetrical (excitatory) synapses in regions exhibiting SPA and in other regions of the same slice lacking SPA in samples derived from three non-epileptic and from three epileptic patients (Figures 5A–D). We measured the length of synaptic active zones in each region, of a total number of 219 and 242 synapses from non-epileptic and epileptic samples, respectively. In several cases, we saw perforated synapses, i.e., two active zones formed by the same terminal to the same postsynaptic element (Figures 5C, D). Perforated synapses have been associated with synapse division (Carlin and Siekevitz, 1983) and with increased synaptic strength and long-term potentiation (Geinisman et al., 1993). Therefore, we analyzed active zones belonging to perforated and non-perforated synapses separately. Altogether, we measured the length of 240 and 281 synaptic active zones in non-epileptic and epileptic tissue, respectively. Overall, the average length of the excitatory synaptic active zones was similar in non-epileptic (0.235 ± 0.006 μm) and in epileptic (0.222 ± 0.005 μm) tissue (Mann–Whitney U test, *p* > 0.05). However, synapses were longer in non-epileptic compared to epileptic tissue in regions generating SPA (for values see Table 6 and Figure 5H, Kruskal–Wallis test, *p* = 0.03).

**TABLE 6:** Length of the active zones of excitatory synapses in the human neocortex, in relation to SPA generation and epilepsy.

	Non-epileptic			Epileptic		
	**Total**	**SPA**	**No SPA**	**Total**	**SPA**	**No SPA**
Number of synapses examined	219	115	104	242	117	125
Number of active zones measured	240	126	114	281	133	148
Length of synaptic active zones	0.235 ± 0.006 0.218 [0.164–0.277]	0.241 ± 0.008 0.224 [0.172–0.296]*	0.228 ± 0.009 0.215 [0.159–0.272]	0.222 ± 0.005 0.201 [0.155–0.270]	0.219 ± 0.009 0.196 [0.140–0.284]*	0.224 ± 0.007 0.218 [0.167–0.259]
Significances		Different from epileptic SPA, *p* = 0.03				

Significances: *p < 0.05, two-way ANOVA multiple comparisons *post-hoc* test.

The ratio of perforated synapses relative to the total number of synapses was higher in epileptic (28.8%) than in non-epileptic (20.4%) samples (Figure 5F, two-sided Chi-square test, *p* = 0.027). As the age range of our epileptic patients is considerably lower than that of the non-epileptic patients, we made a regression analysis to assess the effect of age on the ratio of perforated synapses. The number of perforated synapses decreases with age in the monkey hippocampus (Hara et al., 2011), and thus, we expected lower numbers of perforated synapses in the aged non-epileptic patients, the regression analysis showed that age is not a significant predictor of the ratio of perforated synapses (*p* = 0.363, Figure 5G), however, the status of epilepsy is (*p* = 0.042). The synaptic length of perforated synapses was significantly lower than that of non-perforated ones, both in non-epileptic and epileptic samples (Table 7, Kruskal–Wallis test with Dunn’s follow-up test, *p* < 0.0001). Additionally, in regions generating SPA non-perforated synapses were longer in non-epileptic than in epileptic tissue, while no difference was found between perforated synapses, or in regions lacking SPA (Table 7 and Figure 5H, Kruskal–Wallis test with Dunn’s follow-up test, *p* = 0.0335). To exclude the possibility that perforated synapse ratio alterations are partly due to age mismatch between the epileptic and control group, instead of the condition of epilepsy, we performed multiple regression analysis, having the age and epilepsy status as independent variables and perforated synapses as the dependent variable (Figure 5G). The results show that age is not a significant predictor of the ratio of perforated synapses (*p* = 0.363), however, the status of epilepsy has been, on the contrary (*p* = 0.042). This may indicate that synaptic changes are more likely to be attributed to epilepsy than to age.

**TABLE 7:** Length of synaptic active zones of perforated and non-perforated synapses in relation to epilepsy and SPA generation.

	Type of synapse		Length of synaptic active zone	Significances
Non-epileptic	Non-perforated	Total	0.24 ± 0.007 0.23 [0.17–0.30]*	Different from non-epileptic perforated, *p* < 0.0001
		SPA	0.25 ± 0.009 0.24 [0.18–0.31]^#^	Different from epileptic non-perforated, SPA, *p* = 0.0335
		No SPA	0.24 ± 0.01 0.23 [0.16–0.29]	
	Perforated	Total	0.17 ± 0.008 0.17 [0.13–0.20]*	
		SPA	0.19 ± 0.01 0.18 [0.14–0.22]	
		No SPA	0.16 ± 0.01 0.16 [0.11–0.19]	
Epileptic	Non-perforated	Total	0.24 ± 0.007 0.21 [0.16–0.29]^&^	Different from epileptic perforated, *p* < 0.0001
		SPA	0.23 ± 0.01 0.20 [0.14–0.29]^#^	
		No SPA	0.24 ± 0.01 0.23 [0.2–0.28]	
	Perforated	Total	0.17 ± 0.008 0.16 [0.11–0.19]^&^	
		SPA	0.17 ± 0.01 0.15 [0.1–0.19]	
		No SPA	0.18 ± 0.009 0.17 [0.12–0.22]	

*Length of perforated vs. non-perforated synapses is significantly different in non-epileptic samples.

^#^Length of non-perforated synapses is significantly different in epileptic vs. non-epileptic samples, in regions generating SPA.

^&^Length of perforated vs. non-perforated synapses is significantly different in epileptic samples.

Next, we examined the target elements of the excitatory synapses. They terminated exclusively on dendritic spines, and dendritic shafts, we did not observe cell body or axon initial segment as postsynaptic target element. In a few cases we could not determine the postsynaptic element, and these profiles were classified as unidentified elements (see section “2. Materials and methods”). Significantly higher numbers of spines were seen among the target elements of excitatory axon terminals in epileptic compared to non-epileptic tissue (Table 7, two-sided Chi-square test, *p* = 0.027). We did not find differences in the target distribution, when comparing regions generating and lacking SPA (non-epileptic tissue, Chi-square test, *p* = 0.787, epileptic tissue, Fisher’s exact probability test, *p* = 0.96). More than one bouton terminating on the same postsynaptic target was seen quite rarely. Dendritic shafts received multiple synapses in 10/219 cases (4.6%) in the non-epileptic and in 6/242 (2.5%) cases in the epileptic neocortex (*p* > 0.05, Chi-square with Yates’s correction). Two axon terminals giving synapses to the same spine were observed in 2/219 (0.9%) and in 7/242 (2.9%) cases in non-epileptic and epileptic samples, respectively (*p* > 0.05, Fisher’s exact test).

The length of the synaptic active zones on spines (0.21 ± 0.005; 0.19 [0.15–0.26]) was significantly lower than that on dendrites (0.24 ± 0.007; 0.22 [0.17–0.29], Kruskal–Wallis test with Dunn’s follow-up test, *p* = 0.0021, Figure 5E). We also examined synaptic lengths in relation to SPA and epilepsy. The difference in synaptic lengths regarding their postsynaptic targets comes from the differences seen only in epileptic, but not in non-epileptic tissue (Figure 5H, Kruskal–Wallis test with Dunn’s follow-up test, *p* = 0.0248). We found that in regions initiating SPAs, synaptic lengths on spines were significantly lower in epileptic compared to non-epileptic tissue (for values see Table 8, Kruskal–Wallis test with Dunn’s follow-up test, *p* = 0.0035, Figure 5I). Furthermore, in regions lacking SPA, synaptic lengths on spines were lower than on dendrites, only in tumor tissue (Kruskal–Wallis test with Dunn’s follow-up test, *p* = 0.0026, Figure 5I).

**TABLE 8:** Target elements of excitatory synapses in the human neocortex, in relation to epilepsy and SPA generation.

		Number of examined active zones	Spine		Dendritic shaft		Un-identified
			***N* (%)**	**Length of active zone**	***N* (%)**	**Length of active zone**	***N* (%)**
Non-epileptic	Total	240	128 (53.3)*	0.22 ± 0.008 0.20 [0.15–0.27]	93 (38.8)	0.25 ± 0.01 0.23 [0.17–0.30]	19 (7.9)
	SPA	126	65 (51.6)	0.24 ± 0.01; 0.23 [0.16–0.3]^$^	51 (40.5)	0.23 ± 0.01; 0.22 [0.17–0.26]	10 (7.9)
	No SPA	114	63 (55.3)	0.20 ± 0.01; 0.19 [0.15–0.25]^#^	42 (36.8)	0.26 ± 0.01; 0.25 [0.18–0.33]^#^	9 (7.9)
Epileptic	Total	281	187 (66.5)*	0.21 ± 0.007 0.19 [0.14–0.24]^&^	85 (30.2)	0.23 ± 0.01 0.19 [0.16–0.30]^&^	9 (3.2)
	SPA	133	89 (66.9)	0.20 ± 0.01; 0.18 [0.13–0.23]^$^	41 (30.8)	0.24 ± 0.01; 0.22 [0.13–0.35]	3 (2.3)
	No SPA	148	98 (66.2)	0.21 ± 0.009; 0.21 [0.15–0.25]	44 (29.7)	0.23 ± 0.01; 0.22 [0.17–0.27]	6 (4.1)

*Non-epileptic is significantly different from epileptic (two-sided Chi-square test, *p* = 0.0027).

^$^In regions with SPA synaptic lengths on spines are different in non-epileptic and epileptic tissue (Kruskal–Wallis test with Dunn’s follow-up test, *p* = 0.0035).

^#^In regions without SPA synaptic lengths on spines and dendrites are different only in tumor tissue (Kruskal–Wallis test with Dunn’s follow-up test, *p* = 0.0026).

^&^In epileptic tissue synaptic length is different if the postsynaptic target is spine or dendrite (Kruskal–Wallis test with Dunn’s follow-up test, *p* = 0.0248).

In summary, excitatory synapses have been changed in epilepsy. More perforated synapses were seen in epileptic than in non-epileptic tissue, and together with this, the synaptic length decreased, but only in regions generating SPA. The proportion of spines as postsynaptic targets increased in epilepsy, and synaptic length on spines was lower than that on dendrites. However, this latter phenomenon was also visible in non-epileptic tissue, in regions lacking SPA.

## 4. Discussion

### 4.1. The role of glutamate receptors in the generation of synchronous population activity

Spontaneous SPA emerged in human neocortical slices (Tóth et al., 2018; Kandrács et al., 2019; Hofer et al., 2022). As SPA is generated in slices derived from both epileptic and non-epileptic patients, it is considered to be a non-epileptic synchrony (Hofer et al., 2022). The network properties (recurrence frequency, LFPg and MUA amplitudes) were similar in epileptic and non-epileptic samples [present study and Tóth et al. (2018)], such as the initiation mechanisms (Hofer et al., 2022). Both excitatory and inhibitory circuits participate in the generation of SPA (Tóth et al., 2018; Hofer et al., 2022), and it seems that perisomatic inhibition does not have a leading role in this process (Tóth et al., 2021). In this study, we examined the role of glutamatergic signaling in the generation of SPA. The application of the AMPA/kainate type of GluR antagonist NBQX reversibly abolished the emergence of SPA. AMPA/kainate GluRs are essential in transmitting excitation in the vast majority of excitatory synapses, all over the neocortex, and thus, are likely to participate in all kinds of synchrony generation (Conti and Weinberg, 1999). Although the different subunits of the AMPA/kainate GluRs are differently up- or down-regulated in epilepsy (DeFelipe et al., 1994; Gonzalez-Albo et al., 2001), their ubiquitous presence ensures their pivotal role in the emergence of SPA. On the other hand, NMDA-R blockade with APV showed very little effect on the generation of SPA, it decreased the recurrence frequency in epileptic, but not in non-epileptic tissue. This contrasts to the findings of a previous study where the application of APV had no effect on the generation of spontaneous sharp waves in neocortical slices derived from epileptic patients (Köhling et al., 1998). Their experimental circumstances (slice handling, chamber type, composition of the bathing solution, and temperature) slightly differed from ours, which might be behind this discrepancy. However, it has to be mentioned that the elevation of Mg^2+^ concentration in the superfusate – which accentuates the blockade of the synaptic transmission mediated by NMDA receptors – reduced or even suppressed the spontaneous activity in a few slices (Köhling et al., 1998). The decrease in recurrence frequency of SPAs upon APV application observed only in slices derived from epileptic patients might result from the modified receptor expression in epilepsy (Mathern et al., 1998). Increased numbers of NMDA-R positive neurons were shown in the epileptogenic neocortex both in supragranular (layers II/III) and infragranular (layer VI) layers, compared to non-epileptogenic samples (Gonzalez-Albo et al., 2001), although with a slightly decreased overall expression of the NMDA receptor subunits (Wyneken et al., 2003). As the pharmacological agent can act on higher numbers of cells, this might cause a stronger effect (i.e., a greater, and therefore significant decrease) on the recurrence frequency of SPAs.

### 4.2. Activity of different cell types and the effect of glutamate receptor blockade

Analyzing single unit activities in the presence of NMDA and AMPA/kainate receptor showed that antagonists did not alter the overall firing rate or burstiness of the different excitatory and inhibitory cell types, however, a modest decrease in firing rate took place in the course of the pharmacological experiment in non-epileptic tissue. Analyzing various cell types separately, we must note that in the epileptic tissue, a considerable number of INs started to fire when both NMDA and AMPA/kainate GluRs were blocked. Several IN types were shown to be capable of sustained firing when glutamatergic synaptic transmission was blocked, and thus be able to maintain synchronous activities such as theta oscillation (Blatow et al., 2003) or epileptic seizure (Levesque et al., 2016). We can only speculate that the above mentioned INs detected in our epileptic samples might show this excitation-independent firing feature and have a leading role in generating epileptic synchronies.

Intrinsically bursting feature of neocortical pyramidal cells has been associated to epilepsy (Prince and Wong, 1981), and was shown to participate in the initiation of interictal spikes both in humans (Hofer et al., 2022) and animal models (Connors, 1984). In previous studies, application of the non-NMDA receptor antagonist CNQX (Hwa and Avoli, 1992) or the NMDA receptor antagonist APV (Avoli and Olivier, 1989) blocked the burst firing of human neocortical pyramidal cells in intracellular records. We could not confirm these results, since IB-PCs could be identified based on their autocorrelogram both in APV (*n* = 7 out of 23 PCs altogether, see also Figure 2B) and in APV + NBQX solution (*n* = 6 out of 22 PCs altogether) and the burstiness index of all PCs was also comparable to values found in control conditions. We cannot find a reasonable explanation for the inconsistency concerning blockade of NMDA receptors. The experimental conditions (temperature, slice thickness, interface chamber, composition of the bath solution, and APV concentration) were comparable to ours, such as the location and number of the recorded cells (middle and infragranular layers, 6 vs. 7 neurons). Differences in the results concerning antagonizing non-NMDA receptors might be explained by either the different antagonist used (CNQX vs. NBQX), or the low number of bursting cells examined in the intracellular study (*n* = 3). Although blockade of GluRs did not modify the burstiness of the neurons in general, combining all cells in all pharmacological conditions resulted in a significantly higher burstiness index in epileptic than in non-epileptic tissue. On the other hand, during application of APV + NBQX the burstiness of IB-PCs was lower in epileptic compared to non-epileptic tissue. This contrasts and supports our previous study where the burstiness of all cells – and especially that of PCs – in physiological conditions was lower in epilepsy (Hofer et al., 2022), and is possibly explained either by the difference in the pharmacology or by the bias resulting from substantially lower cell numbers of this study. Furthermore, IN in epileptic samples showed a decreased burstiness in APV + NBQX bath compared to control conditions, which might suggest that blockade of the GluRs affects the firing properties of INs in a different way than that of other cell types.

Intrinsically bursting PCs were overrepresented in mono- and polysynaptic connections detected based on the cellular crosscorrelograms. This may be attributed to the characteristics of the firing pattern itself, since AP bursts are more likely to evoke APs in the postsynaptic neuron (Csicsvári et al., 1998). As in the hippocampus during sharp-wave ripple complexes in animal models (Buzsáki, 2015), this might be a powerful way to transfer information in the human neocortex as well. However, we cannot exclude the possibility that this observation is biased. Neuronal discharge coming from burst firing might sum up more easily and therefore IB-PCs could pass the criteria of having sufficient APs to be included in the synaptic connection analysis.

### 4.3. Epileptic reorganization in the human epileptic neocortex

We examined the glutamatergic circuitry and its changes in epilepsy in the human neocortex, at network, cellular and subcellular levels. Neuron loss related to epilepsy has been described in the human neocortex (Thom et al., 2005; Tóth et al., 2018), however, our results indicate that the SMI-32-positive projecting pyramidal neuron population is not affected by this cell loss. The firing rate of neurons located in the granular layer highly increased in epilepsy. This change may be attributed to alterations in the laminar microstructure of the human neocortex, due to epileptic reorganization. Higher density of synapses characterizes supragranular and granular layers (Marco and DeFelipe, 1997). Furthermore, axon terminals in layer IV had a remarkably large pool of releasable synaptic vesicles, which may enable these terminals to sustain high-frequency transmission and amplify input signals (Yakoubi et al., 2019). The increased excitation provided by these special axon terminals together with the selective loss of certain interneurons might result in releasing layer IV cells from inhibition, and thus permitting a higher firing rate. Parvalbumin-positive perisomatic inhibitory cells may be good candidates, as they are very efficient in controlling the synaptic output of their targets (Miles et al., 1996), and their number was demonstrated to be decreased in the human epileptic neocortex (DeFelipe et al., 1993; Tóth et al., 2018). This has to be verified in a future study.

Although modifications related to epilepsy were found on every level, the most prominent changes characterized the subcellular, i.e., the synaptic levels. In our previous study we showed that the density of excitatory synapses was enhanced in the epileptic compared to the non-epileptic human neocortex (Tóth et al., 2018). Now, we demonstrated that higher numbers of perforated synapses characterized the epileptic tissue, with a lower synaptic length of the non-perforated synapses. The postsynaptic targets of the excitatory axon terminals have also been changed in epilepsy, more synapses were found to terminate on spines, together with a decreased length of the synaptic active zones on the spines, compared to non-epileptic tissue. The size of the synapses (i.e., length of the active zones) was higher in synapses terminating on dendrites than on spines: this was detectable in both non-epileptic and epileptic tissue.

All these changes suggest that an intensive reorganization takes place in the human epileptic neocortex, concerning both the presynaptic and the postsynaptic compartments. Modifications in the number of perforated synapses and in the size of the synapse (i.e., the active zone) are linked to the changes on the presynaptic side, whereas alterations in the targets indicate that the postsynaptic neurons are also involved in the reorganization. Perforated synapses are thought to be an intermediate form during the process of synapse division (Carlin and Siekevitz, 1983), turnover (Nieto-Sampedro et al., 1982) and their numbers increased during lesion (Jones, 1999) or epilepsy induced axonal sprouting (Frotscher et al., 2006). Furthermore, the presence of perforated synapses was associated with synaptic plasticity (Geinisman et al., 1993) resulting from several forms of increased synaptic activity, such as for example visual training (Vrensen and Cardozo, 1981) or long-term potentiation (Geinisman et al., 1991; Harris et al., 2003), a form of synaptic plasticity that is implicated in learning and memory (De Roo et al., 2008). Thus, the increased number of perforated synapses in human epilepsy indicates that an excess neuronal activation took place in the neocortical excitatory circuits, suggesting that the observed modifications might be the result of previous seizures and interictal spiking activity in the epileptic brain. The epileptic hyperactivation of the excitatory cells most probably gave rise to axonal growth and synaptic division, which are the basic anatomical processes of epileptic reorganization. Furthermore, differences have been shown in the expression of AMPA and NMDA receptors between perforated and non-perforated synapses (Ganeshina et al., 2004). Perforated synapses were all immunopositive for AMPA receptor and contained significantly higher number of NMDA receptors than non-perforated ones. This phenomenon might contribute to our finding that antagonizing the NMDA receptors significantly decreased the recurrence frequency of SPAs in epileptic – but not in non-epileptic – samples having higher proportion of perforated synapses.

Modifications in the size of the synapses can also indicate axonal growth and sprouting (for review see Kuwajima et al., 2013), and changes in the efficacy of synaptic transmission (Holderith et al., 2012). Higher numbers of AMPA and NMDA GluRs in the postsynaptic density are tightly correlated to a larger area of the synaptic active zone (Takumi et al., 1999), and as a consequence, to a larger postsynaptic response (Harris and Stevens, 1989; Nimchinsky et al., 2004). Excitatory axon terminals were shown to be larger in the CA1 region of the human epileptic hippocampus (Wittner et al., 2002; Witcher et al., 2010), although with a smaller size of the postsynaptic density (Witcher et al., 2010). Similar to the hippocampus, in the neocortex of epileptic patients the active zone length of the neocortical excitatory synapses decreased: both non-perforated synapses and boutons terminating on spines had smaller active zone, than their counterparts in non-epileptic tissue. This phenomenon might be linked to the changes in the ionotropic GluRs observed in epilepsy. Reduction of cell surface AMPA receptors were observed in various brain regions in epileptic patients with focal and generalized-onset seizures (Eiro et al., 2023). The overall NMDA receptor expression was also downregulated in the human epileptic neocortex (Wyneken et al., 2003), and the NMDA receptors were shown to be redistributed in the synapse during epileptogenesis, expressing a decrease in the postsynaptic density and an increase in the extrasynaptic and presynaptic membranes (Frasca et al., 2011). Synapses with smaller active zones containing less GluRs might be a compensatory mechanism to reduce the excess excitation provided by the increased numbers of excitatory boutons during epilepsy. However, a less probable hypothesis cannot be excluded, i.e., synapses with smaller active zone might represent the higher number of dividing synapses, which did not reach their final size yet.

Epileptic reorganization involves modifications in cellular morphology as well, comprising dendritic distortion and spine loss (for review see Swann et al., 2000). This results from the excess excitation provided by seizures and interictal activity, through the prolonged activation of NMDA receptors, as shown in case of dentate granule cells (Isokawa and Levesque, 1991) and neocortical pyramidal cells (Avoli and Olivier, 1989) of epileptic patients. This could imply that axons growing during epileptic synaptic reorganization would contact other cellular compartments than spines (Wittner et al., 2002), or that axon terminals converge on the same spines (Witcher et al., 2010), as in the hippocampal CA1 region derived from epileptic patients with hippocampal sclerosis. On the contrary, we saw more spines among the postsynaptic targets of excitatory terminals in the epileptic neocortex, together with an unchanged ratio of spines receiving multiple synapses, compared to non-epileptic tissue. This suggests that sprouting excitatory axons do not form contacts randomly, but they have a special target selection for spines during epileptic reorganization in the human neocortex.

## 5. Conclusion

The main findings of this study are that glutamatergic signaling through NMDA receptors participate in synchrony generation in the human epileptic neocortex at a higher degree than in non-epileptic samples. The firing rates of the different excitatory and inhibitory cell types did not change during GluR antagonization, but neurons in the granular layer (L4) showed a significantly higher firing rate in epileptic compared to non-epileptic tissue. Intrinsically bursting pyramidal cells were more likely to participate in mono- and polysynaptic connections than other cell types. Excitatory synaptic junctions were altered in epilepsy: shorter synaptic active zones, higher proportion of perforated synapses and more spines among the postsynaptic targets characterized the epileptic samples. Both the electrophysiological and the anatomical data suggest that the excitatory system is altered in epilepsy. This mainly affects the NMDA-dependent glutamatergic signaling and the microstructure of the neocortical neurons attributed to excitatory synaptic transmission. Our findings about the modified excitatory circuitry support the hypothesis that the perturbed balance of excitation and inhibition contributes to the generation of epileptic activity in the human neocortex.

## Data availability statement

The raw data supporting the conclusions of this article will be made available by the authors, without undue reservation.

## Ethics statement

The studies involving human participants were reviewed and approved by the Regional and Institutional Committee of Science and Research Ethics of the Scientific Council of Health. The patients/participants provided their written informed consent to participate in this study.

## Author contributions

RB, LW, and IU contributed to the conception and the design of the study. KT and LW acquired the data. KT, ET, NE, and LW analyzed the anatomical data. RB analyzed the electrophysiological data and performed the statistical analysis. LEr, LEn, and AB provided the human tissue. DF and IU provided the neurological data. RB and LW wrote the first draft of the manuscript. ET and KT wrote sections of the manuscript. All authors contributed to manuscript revision, read, and approved the submitted version.
